# Roles of Myeloid-Derived Suppressor Cell Subpopulations in Autoimmune Arthritis

**DOI:** 10.3389/fimmu.2018.02849

**Published:** 2018-12-04

**Authors:** Min Li, Dongwei Zhu, Tingting Wang, Xueli Xia, Jie Tian, Shengjun Wang

**Affiliations:** ^1^Department of Laboratory Medicine, The Affiliated People's Hospital, Jiangsu University, Zhenjiang, China; ^2^Department of Immunology, Jiangsu Key Laboratory of Laboratory Medicine, School of Medicine, Jiangsu University, Zhenjiang, China; ^3^Department of Laboratory Medicine, Affiliated Wuxi People's Hospital of Nanjing Medical University, Wuxi Children's Hospital, Wuxi, China

**Keywords:** autoimmune arthritis, myeloid-derived suppressor cells, PMN-MDSCs, MO-MDSCs, immunosuppression

## Abstract

Emerging evidence suggests the promise of the use of myeloid-derived suppressor cells (MDSCs) in inflammatory disorders based on their unique immune-intervention properties. However, the roles of MDSCs in autoimmune arthritis are not completely understood. Indeed, their immunosuppressive functions in arthritic conditions remain controversial, with heterogeneity among MDSCs and differential effects among subpopulations receiving much attention. As a result, it is necessary to determine the roles of MDSC subpopulations in autoimmune arthritis to clarify their diagnostic and therapeutic potential. Interestingly, in the inflammation niche of autoimmune arthritis, each MDSC subpopulation can exhibit both alternatives of a given characteristic. Moreover, polymorphonuclear MDSCs (PMN-MDSCs) are likely to be more suppressive and stable compared with monocytic MDSCs (MO-MDSCs). Although various important cytokines associated with the differentiation of MDSCs or MDSC subpopulations from immature myeloid precursors, such as granulocyte colony-stimulating factor (G-CSF), have been largely applied in external inductive systems, their roles are not entirely clear. Moreover, MDSC-based clinical treatments in rheumatoid arthritis (RA) continue to represent a significant challenge, as also reported for other autoimmune diseases. In this review, we describe the effects and actions of MDSC subpopulations on the development of autoimmune arthritis and analyze several types of MDSC-based therapeutic strategies to provide comprehensive information regarding immune networks and a foundation for more effective protocols for autoimmune arthritis.

## Introduction

Rheumatoid arthritis (RA) is a chronic systemic autoimmune disease characterized by multiple invasive and symmetrical joint inflammation and organic dysfunction (1). Abnormal interactions among inflammatory cells, such as Th17 cells, B cells, macrophages, dendritic cells (DCs), fibroblasts and osteoclasts, in inflamed tissues significantly contribute to the occurrence and evolution of autoimmune arthritis, mainly through molecular docking or the release of active products (2). Among CD4^+^ T cell subpopulations, CD4^+^CD25^+^Foxp3^+^ regulatory T cells (Tregs) represent a limited valuable subpopulation in RA treatment, and the balance of Th17 cells and Tregs is an important factor associated with the development of inflammation (1, 3). Myeloid-derived suppressor cells (MDSCs) comprise a group of highly heterogeneous cells derived from immature myeloid progenitors that are typically divided into two subpopulations: polymorphonuclear (PMN-MDSCs) and monocytic (MO-MDSCs) MDSCs (4–6). Although numerous reports have demonstrated the potent immunosuppression effects of MDSCs under abnormal conditions, the roles of MDSCs and their subpopulations in autoimmune arthritis remain unclear. As research about MDSCs in arthritis progresses, differences in the roles and actions of MDSC subpopulations have stimulated growing interest among researchers. In this review, we summarize the current research findings of MDSC subpopulations in autoimmune arthritis and evaluate the prospects of their application.

## MDSCs

MDSCs are generally characterized by Gr-1^+^CD11b^+^ status in mice and CD11b^+^CD33^+^ status in humans. In normal mice, MDSCs reside in the bone marrow (BM) and spleen (~20–30 and 2–4%, respectively) (4–6). However, because of their complex compositions, MDSCs can not be completely defined exclusively by combinations of several surface molecules, which might explain part of the functional inconsistencies noted in the same disease models (7). As a result, it is necessary to define MDSC subpopulations that are highly suppressive and further delineate their corresponding immune regulation mechanisms within various local environments.

### MDSC Subpopulations

Most reports involving mice classify MDSCs according to Ly6C and Ly6G expression and Gr-1 composition to define their morphologies and origins (8, 9). PMN-MDSCs and MO-MDSCs are characterized as CD11b^+^Ly6C^+^Ly6G^+^ and CD11b^+^Ly6C^+^Ly6G^−^, respectively (10). To detect the immune function of each subpopulation of MDSCs, some studies further divide Ly6C expression into Ly6C^hi^, Ly6C^lo^, and Ly6C^int^ (11–15). Other markers, such as CD115^+^, CD49^+^, and IL-4R^+^, have also been used to represent highly suppressive MDSC subpopulations. CD115 expression levels correlate with Treg expansion induced by MDSCs (16–18). IL-4R expression is closely associated with CD8^+^T cell inhibition (19), whereas CD49^+^ MDSCs reflect MO-MDSCs suppressive toward both antigen-specific and non-specific T cell proliferation (8, 20–22).

In humans, both PMN-MDSCs and MO-MDSCs from human peripheral blood (PB) inhibit the activation and expansion of autologous T cells. However, the marker profile of MDSC subpopulations is more complex than that in mice. Multiple combination types of surface markers, mostly discussed within specific types of cancer patients, were reported to select or define PMN-MDSCs and MO-MDSCs mainly sourced from human PBMCs (14, 23). Several combinations are selectively listed here, which have been determined from both nuclear morphology and suppressive activities. For PMN-MDSCs, the effective combinations of surface markers including CD11b^+^ CD15^+^ CD33^low^ (24), CD11b^+^ CD14^neg^ CD15^+^ (25), CD11b^+^ CD33^+^ CD14^neg^ CD15^+^ (26), CD11b^+^ CD33^+^ CD66b^+^ VEGFR1^+^ CD14^neg^ CD15^+^ (27), and CD11b^+^ CD33^+^ HLA-DR^neg^ CD14^neg^CD66b^+^CD15^+^ and so on (28), while CD14^+^HLA-DR^neg/low^ (29), CD14^+^IL-4Ra^+^ (30) were the most frequently used for MO-MDSC identifications (31). Bronte V and colleagues reviewed the minimum requirements for definition and characterization of human PMN-MDSCs and MO-MDSCs in peripheral blood mononuclear cells (PBMCs) are, respectively CD11b^+^CD14^−^CD15^+^/CD66^+^ and CD11b^+^ CD14^+^CD15^−^HLA-DR^−/low^ (32). Functionally, increased PD-1 expression on human PMN-MDSCs in multiple sclerosis (MS) was able to partially mediate T cell inhibition (33). High levels of S100 calcium-binding protein A9 (S100A9) on MDSCs were found to be consistent with NOS2 activity, representing a potential marker for human MO-MDSCs (34). These evidences suggest that several surface markers CD11b, CD14, CD15, CD66, and HLA-DR have been considered as applicable surface markers so far for the corresponding purification of PMN-MDSCs and MO-MDSCs. Whether other potential surface markers for human PMN/MO-MDSCs mentioned above such as CD62L^low^, CD66b^hi^, IL-4Ra^+^, PD-1^+^, S100A9^+^ and high levels of vascular endothelial growth factor receptor 1 (VEGFR1) (35) are widely applicable or more effective for the purification of functional PMN/MO-MDSCs need to be further demonstrated.

Importantly, we can conclude that at the present, in the matter of possibly applicable surface markers for the precise purification of highly suppressive MDSC and/or MDSC subpopulations regardless from animal models or humans, most of these candidates were proposed only in specific disease settings; in other words, these markers are not universal and limited to one or several disease backgrounds. For example, expression of CD124 or CD115 on MDSCs was not significantly associated with MDSC-mediated suppression of CD8^+^ T cell responses in ET-4 lymphoma models, which disagreed with what was initially reported for C26-GM tumor cell lines (19, 36). Therefore, more evidence needs to be collected to support the common application of these markers.

### MDSCs in Local Environments

MDSCs expand and accumulate within multiple pathological disorders and regions, such as tumor sites, inflammatory lesions, PB or the spleen, generally due to blockade of normal differentiation from hematopoietic stem cells (HSCs) to mature myeloid cells. Significant increases in MDSCs have also been observed in the PB of patients with cancers (4, 7, 8, 37) as well as multiple infections and autoimmune diseases (9, 38).

The systematic effects of MDSCs are controversial with regard to tumors and within the context of inflammatory disease progression. In general, murine MDSC-mediated immunosuppression involves secretion of active products, such as arginase1 (arg-1), nitric oxide (NO), reactive oxygen species (ROS), and inducible nitric oxide synthase (iNOS), and expression of vital signal molecules, such as programmed cell death protein 1 (PD-1). Effects include the following: direct or indirect suppression of CD4^+^T cell activation, proliferation or migration; cooperation with Tregs; and communication with multiple mature myeloid cells or tumor cells (37, 39). Arg-1, an enzyme that catalyzes hydrolysis of L-arginine (40), is more frequently utilized by PMN-MDSCs to block normal signal activation of T cells by directly inhibiting formation of the cyclin D3 (CD3 ζ) and cyclin-dependent kinase 4 complex due to arginine deficiency (41, 42). ROS, including superoxide anion and NO, also contribute to PMN-MDSC-mediated interruption of T cell proliferation (36, 43, 44). iNOS promotes decreased expression of signal transduction, activation of transcription 5 (STAT5) in MO-MDSCs and MHC II in inflammatory cells (36, 45, 46). Moreover, human MDSCs potently inhibit T cell proliferation *in vitro* (47–49). The pro-inflammatory effects of MDSCs are mainly mediated via the promotion of Th17 cell polarization, CD8^+^ T cell activation and their differentiation potentials into mature cells (50–54), which are primarily observed in animal models.

The local environment is one of the most important factors that regulates immune cell functions *in vivo*, and MDSCs are not excepted. On the one hand, different suppressive capacities and functional mechanisms of MDSCs have been demonstrated during disease development. A study of helminth infection reported that MDSCs from early-stage-infected mice were able to induce release of IFN-γ from T cells and inhibit T cell proliferation, and these activities were primarily mediated by NO. At later stages, the induction ability of IFN-γ released from T cells was lost, with arg-1 playing more important roles in the inhibition process than NO (55). Moreover, the role of MDSCs in T cell suppression was found to be reduced after the onset of collagen-induced arthritis (CIA) (14). These variations in MDSC function suggest the important influence of the local environment and are possibly related to the alternative activation phenotypes or variable expansion of MDSC subpopulations. On the other hand, MDSCs located at the periphery and center of lesions exhibit distinct behaviors that are not only related to functional changes in MDSCs themselves (56) but can also be attributed to the maturity or composition of contiguous immune cells involved in distinct local niches. This finding is similar to the discovery that Tregs in the synovial fluid (SF) of RA patients and mice with autoimmune arthritis display more mutual phenotypes and more potent immunosuppression compared with Tregs in PB (57, 58). The differentiation programs of Ly6C^hi^ monocytes can also be switched from anti-inflammatory macrophages to inflammatory DCs within the context of inflammation in the colon (53). Given the wide interactions of immune cells with MDSCs at diseased sites, assessment of the compositions and functional states of immune cells around MDSCs within lesion sites is beneficial for accurate identification of the immune network and the development of more applicable MDSC-based therapies.

## MDSCs in Autoimmune Arthritis

Similarly, the frequencies of MDSCs during the development of autoimmune arthritis are aberrant. Compared with healthy controls, MDSCs were increased in the spleen, paws, BM, and draining lymph nodes (DLNs) (popliteal or inguinal lymph nodes, periarticular lymph nodes) of experimental autoimmune arthritis models (10, 14, 59–63) and in the PB and SF of RA patients (61, 64). Most studies report that the frequencies of both PMN-MDSCs and MO-MDSCs in the spleen of experimental autoimmune arthritis models were decreased at the early stage but increased at the late stage, which might indicate a procession of myelopoiesis and functional roles during immunoregulation. Moreover, it has been reported that the number of circulating MDSCs in RA patients is positively correlated with Disease Activity Scores in 28 Joints (DAS28) and negatively correlated with Th17 cell frequencies.

### Identification of MDSC Subpopulations in Autoimmune Arthritis

In an autoimmune arthritis mouse model, PMN-MDSCs have been identified as CD11b^+^Gr-1^high/+^ or CD11b^+^Ly6G^+^ Ly6C^+/−/int/low^, whereas MO-MDSCs have been identified as CD11b^+^Gr-1^medium^ or CD11b^+^Ly6C^+/high^Ly6G^−^ (Table 1). CCR2, CD62L, and CD115 (14) expression on MO-MDSCs derived from experimental autoimmune arthritis models and tumors has also been reported (67). Low levels of other markers such as MHC II, F4/80, and CD11c on MDSCs are indicative of their myeloid source and immature features (14). Increased CD40 and CD86 expression on MO-MDSCs isolated from CIA mice compared with PMN-MDSCs has been observed, though no significant differences in surface expression of CD80, MHC II, or PD-L1 have been reported (10). In RA patients, MO-MDSCs are commonly marked by CD14^+^HLA-DR^−/low^ (62), whereas PMN-MDSCs are identified by CD11b^+^ CD33^+^ HLA-DR^low/−^ CD14^−^ CD15^+^ (61). CD11b, which is also expressed on neutrophils, monocytes, macrophages and NK cells, is the only common marker of MDSC subpopulations in humans and mice.

**Table 1:** MDSC subpopulations in autoimmune arthritis.

**Source**	**Subpopulation**	**Phenotype**	**Description**	**MDSCs (%)**	**Sample**	**References**
RA patients	PMN-MDSCs	CD11b^+^ HLADR^lo/−^ CD33^+^CD14^−^CD15^+^	Inhibit Ag-specific and non-specific autologous T cell proliferation		SF	(61)
	MO-MDSCs	CD14^+^HLA-DR^−/low^	Increased MO-MDSCs in SF and PB are positively correlated with circulating Th17 cells and RA activity	↑	SF PB	(65)
CIA in C57BL/6	PMN-MDSCs	CD11b^+^Ly6C^low^Ly6G^+^	No inhibition of T cell proliferation and IFN-γ secretion *ex vivo* (from SP)	↑	SP paws	(14)
	MO-MDSCs	CD11b^+^Ly6C^high^Ly6G^−^ CD115^+^CCR2^+^CD62L^+^F4/80^low^	Inhibit T cell proliferation and IFN-γ production *ex vivo* via iNOS but not arg-1 (from SP); promote differentiation of Th17 cells *ex vivo* dependent on IL-1β signaling (from SP)	↑	SP paws	(14)
CIA in DBA/1J	PMN-MDSCs	CD11b^+^Ly6C^+^Ly6G^+^	Alleviate CIA after adoptive transfer by inhibiting T cell proliferation and Th1 and Th17 cell differentiation	Early stage ↓ Late stage ↑	SP	(10)
	PMN-MDSCs	CD11b^+^ Ly6G^+^MHCII (I-Ab) ^low^ CD11c^low^		Early stage ↓ Late stage ↑	SP	(65)
	PMN-MDSCs	CD11b^+^ Gr-1^high^Ly6G^+^ Ly6C^+^CCR2^−^CD115^low^ F4/80^−^CD11c^−^	Slightly promote proliferation of autologous CD4^+^T cells; no effect on B cell proliferation		BM	(13)
	PMN-MDSCs	CD11b^+^Ly6G^+^Ly6C^low^ CD11c^−^	Attenuate joint inflammations after adoptive transfer by inhibiting Th17 cell differentiation and supporting Treg expansion, likely via IL-10		SP	(66)
	MO-MDSCs	CD11b^+^Ly6C^+^Ly6G^−^	No effect after adoptive transfer; inhibit T cell proliferation but no inhibition of Th1 cell differentiation and slightly promote Th17 cell differentiation	Early stage ↓ Late stage ↑	SP	(10)
	MO-MDSCs	CD11b^+^Gr-1^medium^Ly6C^+^F4/80^+^CD11c^low^MHC II(I-Ab) ^low^		Early stage ↓ Late stage ↑	SP	(65)
	MO-MDSCs	CD11c^−^CD11b^+^Ly6G^−^ Ly6C^high^	Attenuate joint inflammation more potently after adoptive transfer compared with PMN-MDSCs by inhibiting Th17 cell differentiation and supporting Treg cell expansion, likely mediated by IL-10 but not arg-1, iNOS or TGF-β		SP	(66)
	MO-MDSCs	CD11b^+^Ly6C^high^ Ly6G^−^ Gr-1^mod^ CCR2^+^CD115^+^ F4/80^low^CD11c^−^	Inhibit T cell proliferation dependent on iNOS and IFN-γ and independent of IL-17; inhibit autologous B cell proliferation and antibody production via iNOS and PGE_2_ in a contact-dependent manner		BM	(13)
AA in CD-1	PMN-MDSCs	Gr-1^high^Ly6G^high^ CD49^+^SSC^low^	Exhibit greater suppression of T cell proliferation with higher ROS production	↑	SP	(20)
	MO-MDSCs	Gr-1^dim^Ly6G^−^		↑	SP	(20)
PGIA in BALB/c	PMN-MDSCs	Ly6G^hi^Ly6C^int/lo^	Inhibit DC maturation and Ag-specific T cell proliferation via ROS and iNOS	>90% in SF	SF SP	(11)
	MO-MDSCs	Ly6C^hi^Ly6G^neg/low^		~1% in SF ~4% in SP	SF SP	(11)

*RA, rheumatoid arthritis; CIA, collagen-induced arthritis; PGIA, proteoglycan-induced autoimmune arthritis; AA, adjuvant arthritis; PMN-MDSCs, polymorphonuclear myeloid-derived suppressor cells; MO-MDSCs, monocytic myeloid-derived suppressor cells; DCs, dendritic cells; Tregs, regulatory T cells; iNOS, inducible nitric oxide synthase; PGE2, prostaglandin E2; ROS, reactive oxygen species; arg-1, arginase1; SP, spleen; SF, synovial fluid; PB, peripheral blood; BM, bone marrow*.

It was suggested that phenotypes of MDSC subpopulations vary within the context of evolving microenvironments, and similar phenotype characteristics have been noted for other precursors and mature myeloid cells. For example, inflammatory monocytes are characterized by CD11b^+^Ly6C^high^Ly6G^−^CD115^+^MHC II ^−^F4/80^+^CD11c^−^, which is similar to MO-MDSCs (8, 14, 17, 18). In addition, PMN-MDSCs with variable phenotypes exhibit characteristics similar to those of CD11b^+^Ly6G^+^Ly6C^low^ F4/80^−^CD11c^−^ granulocytes (8). As a result, both surface markers and key immune functions are necessary for identifying MDSC subpopulations. Moreover, standard methods for isolating MDSC subpopulations must be developed to facilitate comparison among studies.

### Roles of MDSCs in Autoimmune Arthritis

Two different effects of MDSCs on immune responses during the development of autoimmune arthritis have been observed. Attenuation of arthritis has been attributed to MDSC-mediated disruptive effects on “vicious cycles of inflammation,” including suppression of T cell proliferation and the unfavorable impact on the responses of pro-inflammatory cells, such as CD8^+^T cells, Th17 cells and DCs. This mainly occurs via the mediators mentioned above, including arg-1, iNOS, and IL-10 (7, 9). iNOS plays a vital role in MDSC-mediated T cell suppression and more effectively inhibits antigen (Ag)-specific T cell proliferation compared with arg-1, as observed in mice with proteoglycan-induced autoimmune arthritis (PGIA). These data indicate the significant therapeutic potential of MDSCs for RA, which is consistent with their contributions to piperlongumine-attenuated CIA (60, 62). Undesirable pro-inflammatory effects of MDSCs have also been reported in animal models of RA and appear to facilitate responses by Th17 (65) and B (15) cells.

MDSC activities in distinct organs, such as the BM, PB, spleen or synovial membranes, have been reported (Table 2). Interestingly, in the spleens of CIA mice, MDSCs exhibit both inhibitory and promoting effects on Th17 cell differentiation, and it is unclear how these results can be reconciled. Nonetheless, the latter exhibited a stronger correlation with MO-MDSCs, in which IL-1 functions as a potentially critical supporter (14, 62), as previously demonstrated in experimental autoimmune encephalitis (52). MDSCs from the PB of RA patients also exhibit potential inhibitory activity against Th17 cell accumulation, and other reports have demonstrated a positive correlation between MDSC-PB and B cell proliferation. Further relationships between MDSC subpopulations and B cells in autoimmune arthritis are described below (see section MO-MDSC Involvement in Autoimmune Arthritis). More potent suppression of Ag-specific, as opposed to Ag-non-specific, T cell proliferation and responses by MDSCs from the spleen and SF of both RA patients and experimental autoimmune arthritis models have been reported (11, 61). MDSCs isolated from the SF of PGIA mice were also capable of suppressing DC maturation via iNOS and ROS and of inhibiting DC-dependent T cell proliferation with elevated expression of arg-1, NO and ROS. Some MDSCs derived from the BM of CIA mice showed enhanced transformation into mature myeloid cells compared with those obtained from normal mice (59). It is possible that MDSCs in peripheral and original tissues are less suppressive than are those in lymphoid organs and lesion sites, which is correlated with stimulation of chemokines, cytokines, and inflammatory cells, among others.

**Table 2:** Roles of MDSCs at different sites of autoimmune arthritis.

**Source**	**Site**	**Stage**	**Function/frequency *in vivo***	**Function *in vitro***	**References**
RA patients	PB		Frequencies of MDSCs are opposite to circulating Th17 cell numbers and serum levels of arg-1 and TNF-α		(14, 40)
	PB	High activity	Frequencies of MDSCs are positively correlated with disease activities	Promote B cell proliferation	(64)
	SF			More suppressive in Ag-specific as opposed to Ag-non-specific T cell proliferation	(61)
PGIA	SF	At the peak		Suppress DC maturation and DC-dependent T cell proliferation by iNOS and ROS	(11)
CIA	SP	At the peak	Aggravate disease severity by promoting Th17 cell response after adoptive transfer	Inhibit Ag-non-specific T cell proliferation and response by iNOS; enhance Th17 cell differentiation by IL-1β; T cell-suppressive activity of MDSCs was decreased after disease onset	(14)
	SP	At the onset	Promote Th17 cell differentiation and response; no effect on Tregs after depletion No significant effects after depletion but restore arthritis and Th17 cell response after adoptive transfer following the depletion	More suppressive in Ag-specific as opposed to Ag-non-specific T cell proliferation; promote Th17 cell differentiation by IL-1β	(65)
		At the peak			
	SP	At the peak	Attenuate joint inflammations by reduction of Th1 and Th17 cells and increase of Tregs in CIA mice	Support Tregs but inhibit Th17 cell differentiation; decrease Ag-specific T cell proliferation	(66)
	SP	At the peak	Resist spontaneous improvement of CIA by inhibiting Th17 cell and T cell response	Inhibit Ag-non-specific T cell proliferation and response and Th17 cell differentiation	(15)
	BM	At the peak	Differentiate into osteoclasts after adoptive transfer	Less potent inhibition of Ag-non-specific CD4^+^T cell proliferation than normal MDSCs; differentiate into osteoclasts mediated by the IL-1-activated NF-κ B pathway	(59)

*RA, rheumatoid arthritis; CIA, collagen-induced arthritis; PGIA, proteoglycan-induced autoimmune arthritis; PMN-MDSCs, polymorphonuclear myeloid-derived suppressor cells; MO-MDSCs, monocytic myeloid-derived suppressor cells; DCs, dendritic cells; Tregs, regulatory T cells; iNOS, inducible nitric oxide synthase; TNF-α, tumor necrosis factor α; ROS, reactive oxygen species; arg-1, arginase1; NF-κ B, nuclear factor-kappa B; SP, spleen; SF, synovial fluid; PB, peripheral blood; BM, bone marrow*.

For MDSC migration within the context of autoimmune diseases, chemokine receptor 2 (CCR2) functions as a vital chemokine receptor for the migration of MO-MDSCs from the BM into the PB observed in arthritic mice (13). Monocyte chemotactic protein 1 (CCL2) is abundantly present in the synovium of patients with RA (68), whereas MO-MDSCs were absent from the blood, spleen, and lymph nodes of CCR2^−/−^ mice that developed more severe CIA (13). CCL2 on mesenchymal stem/stromal cells (MSCs) also contributes to recruitment of MO-MDSCs to inflammatory sites during MSC-mediated suppression of autoimmunity (69). It has been reported that exposure to IFN-γ markedly upregulates CCR2 expression on MDSCs to retain these cells at inflammatory sites, and MDSCs generated from CCR2^−/−^ mice fail to be mobilized (70). Therefore, IFN-γ and CCR2 may be important mediators during the activation of MDSCs as well as in migration into the periphery during arthritis development. Increased expression of CCR1 on PMN-MDSCs facilitates their entry into the circulation, as observed in systemic lupus erythematosus (SLE) mouse models (71). IL-10 also contributes to the accumulation of PMN-MDSCs in peripheral lymphoid organs during autoimmune disease (72). CXCR2 facilitates MDSC recruitment to allograft sites (73), and IL-6 and tumor necrosis factor alpha (TNF-α) are likely inducers of MDSC accumulation at inflamed tissues to resist aggravation of inflammatory responses (74). Although many other chemokines, such as CCL5, CXCL6, CXCL8, CCL15, CXCL12, have been reported to recruit MDSCs during cancer development (75), these processes in autoimmune diseases need to be further confirmed.

In summary, MDSCs in autoimmune arthritis function as a double-edged sword. More functional regulators of MDSC subpopulations and variability among MDSCs need to be explored to establish adjuvant treatment for MDSC transfer-based therapy targeting these key cytokines or cells.

## Roles of MDSC Subpopulations in Autoimmune Arthritis

Under tumor (2) and transplant antagonism (76–78) conditions, Gr-1^low^Ly6C^high^ MO-MDSCs are generally more suppressive than are Gr-1^high^Ly6C^low^ PMN-MDSCs; however, circulating PMN-MDSCs are more abundant than MO-MDSCs (3:1 ratio) in naive mice (36). In mouse models of RA, PMN-MDSCs also predominate among MDSCs from the PB, DLNs, SF or paws compared with MO-MDSCs (10, 14, 59–63). Additionally, in the pathogenesis of autoimmune arthritis, both PMN-MDSCs and MO-MDSCs exhibit controversial roles and utilize relatively distinguishable pathways to inhibit immune responses, as previously demonstrated for tumors. In contrast, PMN-MDSCs are more suppressive than are MO-MDSCs (Table 1).

### PMN-MDSC Involvement in Autoimmune Arthritis

Previous reports demonstrate that splenic PMN-MDSCs from CIA mice efficiently suppress anti-CD3- or CD28-stimulated T cell proliferation (10). In RA patients, MDSCs isolated from the SF, 95% of which are granulocytic MDSCs, more potently inhibit Ag (allogeneic peripheral blood mononuclear cells)-specific compared with Ag-non-specific autologous T cell proliferation (61). Similarly, SF cells from PGIA mouse models strongly suppress proliferation of DC-activated autologous T cells, but no effect on anti-CD3- or CD28-stimulated T cell proliferation was found (>90% were granulocyte-like MDSCs) (11). As a potent inhibitory cytokine in the synovial membranes of RA patients, IL-10 is associated with T cell anergy (79) and reductions in pro-inflammatory mediators, such as TNF-α and IL-1β (80). IL-10 is also increased in CIA mice and is partly involved in MDSC-mediated T cell inhibition (15). IL-10 also plays an important role during MDSC-mediated attenuation of joint inflammation in CIA mice via Treg cell expansion (66). As a result, inhibition of both Ag-specific and non-specific autologous CD4^+^T cell proliferation is generally involved in PMN-MDSC-mediated suppression of autoimmune arthritis. Regardless, PMN-MDSCs within lesions tend to more specifically execute the former, whereby the release of inhibitory cytokines might represent an important pathway in addition to commonly recognized arg-1- and ROS-mediated effects (Table 1).

PMN-MDSCs also correct the balance of CD4^+^T cell subpopulations. Both Th1 and Th17 cells are vital lymphocytes driving the progression of autoimmune arthritis (1) and significantly related to fibroblast and chondrocyte activation. In pilot clinical trials, the humanized anti-IL-17 monoclonal antibody LY2439821 provided an improved curative effect in RA patients (81). Significantly reduced ratios of IFN-γ^+^ (Th1) and IL-17A^+^ (Th17) cells were observed in the DLNs of CIA mice, and joint inflammation was alleviated after adoptive transfer of PMN-MDSCs (10). This effect in CIA mice was also observed in another study in which PMN-MDSCs promoted Treg cell expansion after injection *in vivo* (66). These results suggest the promising potential of PMN-MDSCs to correct the imbalance in CD4^+^T subpopulations as well as the vicious cycle in the synovial milieu of autoimmune arthritis.

Moreover, PMN-MDSCs efficiently inhibit DC maturation in mouse models of RA. DCs are the predominant antigen-presenting cells and function as an important stimulator in the attraction and subsequent activation of Th1, Th2, and Th17 cells in RA pathogenesis (82). *In vitro*, SF MDSCs potently suppressed DC maturation in a non-cell contact-dependent manner, as demonstrated by reduced expression of DC maturation surface markers CD86 and MHCII (11) and reduced maturation characteristics of SF-isolated DCs compared with those derived from the BM (11). Arg-1 plays vital roles in this process, which is also observed in other autoimmunity diseases (38, 83, 84) and tumors (23, 37). As a result, PMN-MDSC-mediated inhibition of the inflammation generated by monocyte-macrophage lineage cells in inflamed joints might explain such improvement in the signs and symptoms of autoimmune arthritis.

Nonetheless, the pro-inflammatory potential of PMN-MDSCs to develop into neutrophils should not be ignored. Levels of granulocyte colony-stimulating factor (G-CSF), a key factor in neutrophil development and recruitment (85), are significantly increased in the SF of RA patients and correlate positively with RA disease severity (86). Given the extraordinary number of neutrophils and PMN-MDSCs in the SF of RA patients (87, 88) and the destructive ability of neutrophils in joints (89), it is important to consider whether PMN-MDSCs consistently remain in a steady immunosuppressive state or grow and mature under the presence of factors, such as G-CSF, within inflamed joints to transform into neutrophils and promote arthritis development.

### MO-MDSC Involvement in Autoimmune Arthritis

The therapeutic value of MO-MDSCs in autoimmune arthritis is primarily mediated via inhibition of CD4^+^T cells and B cells. CD11b^+^Ly6C^high^Ly6G^−^ monocytes isolated from the BM are associated with a significant reduction in joint damage through effective suppression of CD4^+^T cell proliferation *in vitro*, which is dependent on iNOS and IFN-γ. These MO-MDSCs also inhibit autologous B cell responses via iNOS and prostaglandin E2 (PGE2) in a contact-dependent manner (13). Interestingly, a recent study of RA patients demonstrated that peripheral MDSCs promote autologous B cell proliferation. Although induction of B cell proliferation may decrease PMN-MDSCs, Crook et al. reported no effect of PMN-MDSCs on B cell proliferation. The differences between these two reports may be attributed to the targeted organs and sources of isolated MDSCs. In the former study, MDSCs were isolated from the BM of CIA mice; in the latter study, MDSCs were isolated from the PB of RA patients. These findings indicate the potentially powerful effect of lesion niches on MDSCs and the decreased suppressive effects of MDSCs obtained from the PB compared with BM, as observed in other diseases (56). Inhibition of B cell proliferation mediated by MO-MDSCs may constitute a self-resistance mechanism in excessively inflamed immune systems, though this hypothesis must be confirmed.

MO-MDSCs potently induce the development of Tregs in various inflammatory disorders, such as type 1 diabetes (T1D), transplantation models (73, 77, 90) and tumors (16, 76). CD14^+^HLA-DR^low/−^ MDSCs isolated from human PB alter the differentiation of monocyte-induced Th17 cells into Foxp3^+^ Tregs by secreting transforming growth factor-β (TGF-β) and retinoic acid (91). MO-MDSCs isolated from the spleen of CIA mice ameliorate joint inflammation by promoting Treg cell expansion and inhibiting Th1 and Th17 cell accumulation (66).

Furthermore, contradictory actions of MO-MDSCs have also been observed in the progression of autoimmune arthritis. Levels of CD14^+^HLA-DR^−/low^ MDSCs in the PB or SF of RA patients were found to correlate with peripheral Th17 levels and RA status (14, 62), and IL-17 and IL-10 serum levels were significantly increased after adoptive transfer of MO-MDSCs into CIA mice. Slight acceleration in Th17 cell differentiation by MO-MDSCs was also observed in external co-culture systems (10). IL-1β is likely an important mediator of this progression (14), as observed in experimental models of SLE (71) and MS (multiple sclerosis) (33, 92). In addition, this effect might partially explain the remission of RA after treatment with an IL-1 antagonist (93). These results suggest that MO-MDSCs and PMN-MDSCs may regulate the balance of Th17 and Tregs in autoimmune arthritis, but whether the effect is beneficial for Th17 cells or Tregs in RA pathogenesis has not yet been determined.

Overall, the results described above do not completely support the notion that the pro-inflammatory effects of MO-MDSCs are mediated through inhibition of Ly6C^+^ classical monocytes. CD14^++^CD16^−^ monocytes (classical monocytes), an important human monocyte subpopulation with CD11b expression, represent a main subpopulation of extravascular tissue monocytes (94, 95). Equivalent monocytes in mice are characterized by CD11b^+^Ly6C^+^CD62L^+^CD43^−^CCR2^+^ or Ly6C^+^CD11b^+^ CD11c^−^F4/80^low^CD64^int^ (96), and these monocytes are found in resting non-lymphoid as well as lymphoid tissues, such as the lung and lymph nodes, under steady states (97). Some markers on MO-MDSCs are co-expressed with classical monocytes, such as F4/80, CD115, CD62L, and CCR2 (8, 14, 62). IL-12 promotes Th1 cell proliferation and maturation, Th17 cell differentiation and B cell activation within the synovial milieu, and IL-12 is predominantly released by classical monocytes. As a result, IL-12 is not produced by classical monocytes, potentially accounting for the pro-inflammatory effects of MO-MDSCs described above.

### Are PMN-MDSCs More Suppressive Than MO-MDSCs in Autoimmune Arthritis?

PMN-MDSCs and MO-MDSCs maintain immune homoeostasis of autoimmune arthritis through distinct effector molecules and pathways. However, it remains unclear whether any subpopulation becomes more suppressive as a result of the inflammatory niche. Adoptive transfer of PMN-MDSCs but not MO-MDSCs effectively relieves joint damage in CIA mice. Moreover, T cell proliferation and responses were reported to be more potently suppressed by PMN-MDSCs than by MO-MDSCs, which likely related to IL-10, TGF-β1, CCR5, and CXCR2 (10). Furthermore, PMN-MDSCs more effectively suppress Th17 cell differentiation than MO-MDSCs do (62). The increased therapeutic efficacy and suppressive potential of PMN-MDSCs were also demonstrated in asthmatic mice, which was likely due to Treg cell differentiation and arg-1 production (98). Granulocyte-macrophage colony-stimulating factor (GM-CSF) has a necessary function in the myelopoiesis of MO-MDSCs with great differentiation potential (99). It is abundant in the SF and synovial membranes of RA patients, which is vital for the activation of macrophages (100). As a result, it is important to determine whether MO-MDSCs are steadily maintained under the effects of cytokines, such as GM-CSF, involved in the inflamed joints of autoimmune arthritis or in secondary lymphoid organs, weakening them or rendering them unable to inhibit CD4^+^T cell immune responses. Consistent with the observation for external cultures of PMN-MDSCs and MO-MDSCs, MO-MDSCs mature and develop into CD11b^+^ DCs or F4/80^+^ macrophages in the presence of GM-CSF (36). These experimental data suggest that compared with MO-MDSCs, PMN-MDSCs likely exert a more suppressive function in the pathogenesis of autoimmune arthritis, as reflected by the strong inhibition of T cell proliferation, induction of Treg cell differentiation and a comparative steady state. In addition, interactions between these two subpopulations have also been reported during MDSC-mediated suppression of T cells, such as the production of peroxynitrite, a more suppressive mediator causing T cell unresponsiveness (44).

## Therapeutic Potential of MDSC-Based Treatment

In humans, therapeutic advances in autoimmune diseases have set up therapeutic efficacy of biologic agents according to the pre-clinical/clinical applications of impressive cytokines and monoclonal antibodies for inhibiting inflammatory cytokines (2). Most of them exhibit effective therapeutic effects within partial autoimmune diseases. However, considering many unexpected side effects and unsatisfied efficacy appeared during clinical treatments such as granulocyte reduction, anemia, myelosuppression, injection site response, limited valid periods and relapses, another strategy based on immunosuppressive cell treatment has exhibited a promising prospect.

### Adoptive Transfer of MDSCs *in vivo*

#### Adoptive Transfer of Directly Isolated MDSCs *in vivo*

To understand deeply the therapeutic value of MDSCs in RA patients, we analyzed partial adoptive transfer experiments of MDSCs and/or MDSC subpopulations into experimental animal models of RA. Some typical cases were filtered (Table 3). The results showed that arthritis was improved after total MDSC transfer via suppression of Th17 and Th1 cell accumulation and responses. However, some reports have also suggested aggravated effects, with increased numbers and enhanced responses of Th17 cells and even presentation of differentiation properties. Moreover, we found that injection points might be an important factor related to MDSC functions during adoptive transfer experiments (Table 3). By comparing the completely opposite results, alleviation and aggravation, by Chunqing Guo et al. and Zhang et al., respectively, we noticed that the injection point was before CIA establishment in the former and after in the latter. This finding indicates that the suppressive functions of transferred MDSCs might be more effective within non-strong inflammatory and complicated local environments. It is possible that distinct inflammatory environments stimulate the development of MDSC subpopulations to different extents. Guo et al. also reported that by promoting Th17 cell differentiation, adoptive transfer of MO-MDSCs prior to model establishment exhibits a pro-inflammatory property. Wang et al. suggested poor amelioration of arthritis after MO-MDSC transfer but effective improvement via PMN-MDSC transfer through inhibition of Th17 cell development. These data support the hypothesis mentioned above that MO-MDSCs tend to promote inflammation during autoimmune arthritis. In addition, it has been reported that the ratios of MO-MDSCs among total MDSCs increase steadily until the peak of arthritis, which is contrary to the observations for PMN-MDSCs (10). As a result, it is likely that the environment after arthritis onset is more suitable for MO-MDSC development with pro-inflammatory functions than for PMN-MDSCs, causing exacerbated symptoms. Moreover, it is also possible that seriously inflammatory local environments render MDSCs more changeable, as analyzed previously, resulting in greater difficulty in their suppression and perhaps promoting pro-inflammatory effects. In summary, MDSCs have the potential to regulate the immune imbalance that occurs in autoimmune arthritis, but the differential functions of MDSC subpopulations need to be elucidated.

**Table 3:** Adoptive transfer experiments using MDSCs and/or MDSC subpopulations in experimental animal models of RA.

**Effect**	**Mechanisms**	**MDSC resources**	**Injection points**	**References**
Amelioration	Suppress Th17 cell development	CIA/SP/MDSCs	Before CIA onset	(15)
Amelioration	Suppress Th17 cell and macrophage accumulation; inhibit release of inflammatory cytokines	CIA/SP/MDSCs	Before CIA and AIA onset	(101)
Amelioration	Suppress Ag-specific T cell response; lower serum antibody levels	Normal mice /BM/induced MDSCs	After PGIA onset	(60)
Amelioration	Suppress Th1 and Th17 cell accumulation in DLNs; inhibit the release of inflammatory cytokines	CIA/SP/PMN-MDSCs	Before CIA onset	(10)
Aggravation	Differentiate into osteoclasts via the NF-κB pathway after injection into normal mice	CIA/BM/ induced MDSCs	Normal mice	(59)
Aggravation	Promote Th17 cell accumulation in DLNs; enhance the Th17 cell response	CIA/SP/MDSCs	After CIA onset	(65)
Aggravation	Promote Th17 cell development	– –/SP/MO-MDSCs	Before arthritis onset	(14)
No significant improvement		CIA/SP/MO-MDSCs	Before CIA onset	(10)

*DLNs, draining lymph nodes; CIA, collagen-induced arthritis; PGIA, proteoglycan-induced autoimmune arthritis; AIA, antigen-induced arthritis; PMN-MDSCs, polymorphonuclear myeloid-derived suppressor cells; MO-MDSCs, monocytic myeloid-derived suppressor cells; BM, bone marrow; NF-κB, nuclear factor-kappa B*.

On account of the low frequency of MDSCs in healthy individuals which is unchangeable and the potential threat of immune rejections, it seems that the autologous application of MDSCs is more rational as the initial source of MDSCs than allogeneic transfer. However, the strategy is difficult to practice partially due to the unestablished perfect judgement methods for MDSC physiological and functional conditions, that is, the uncertainty of MDSC conditions in different RA patients and ambiguity clinical stages. Therefore, for autologous transfer of directly isolated MDSCs for RA treatment, it is necessary to resolve two difficulties in advance: making sure exact markers or methods for judgements of MDSC functional conditions and exploring corresponding optimal cultural systems of MDSCs *in vitro* to keep and strengthen their suppressive functions steadily.

#### Adoptive Transfer of Induced MDSCs in vitro

In addition, we want to emphasize another promising clinical application of MDSCs, namely, the induction of MDSCs within the context of autoimmune arthritis to meet the clinical needs for a large quantity of high-quality stable MDSCs. Some MDSC induction methods have been explored *in vitro* using DCs (102), embryonic stem cells (ESCs) (18), HSCs (18), PB mononuclear cells (103) or other normal myeloid progenitor cells (60, 104–106) via combinations of various cytokines. These methods have demonstrated therapeutic value in mouse models of infectious and autoimmune diseases. The initial/progenitor cells and stimulating cells mentioned above used in researches are mostly directly isolated from healthy individuals, which indicates it is allogeneic sourced MDSCs that act as a promising treatment for RA patients based on the adoptive transfer of induced MDSCs *in vitro*. Moreover, in my view, there is also a strong potential for MDSCs induced from autologous initial/progenitor cells for treatments of RA patients and it is undoubtedly more applicable.

As for the critical induction cytokines for MDSC development, it was reported that macrophage colony-stimulating factor (M-CSF) was specifically important for the differentiation of functional MO-MDSCs, whereas GM-CSF was more beneficial for PMN-MDSC development from mouse embryonic and HSCs (18). These induced MO-MDSCs displayed increased suppression of T cell proliferation and promotion of CD4^+^Foxp3^+^ Treg cell expansion compared with those directly isolated from tumors via NO and IL-10, suggesting the potential value of induced MDSCs. It is worth mentioning here in the matter of Treg cell subpopulations which are potentially synergetic with MDSCs *in vivo* that, besides CD4^+^ Tregs mentioned frequently in this review generally identified by CD4^+^ CD25 ^hi^Foxp3^+^, it is likely that another Treg subpopulation, CD8^+^ Tregs, also act as an outstanding candidate capable of facilitating the suppressive functions mediated by MDSCs. It was widely reported that CD8^+^ Tregs were potently suppressive in tumors (107, 108), infectious diseases (109, 110), graft-vs.-host disease (GVHD) (111, 112) and autoimmune diseases such as SLE (113), MS (114), and experiment allergic encephalomyelitis (EAE) (115) mainly by strong inhibitions of CD4^+^T cell proliferations especially for Th1 cells and the pro-inflammatory cytokine productions via the releases of soluble cytokines or co-stimulator expression. In CIA models, CD8^+^ Tregs also showed a powerful suppression contributing to anti-CD3 mAb-improved arthritis and suggested a more potent capability than CD4^+^ Tregs in the suppression of IL-17 production. Importantly, one report in GVHD revealed a close connection between CD8^+^Foxp3^+^Tregs and MDSCs that the former could be induced to contribute the alleviation of GVHD mediated by MDSCs (109) as well as CD4^+^ Tregs. Whether this process or interactions between these two suppressors is true in RA patients is unclear at the present, but there is no doubt that the relationship between CD8^+^ Tregs and MDSCs is worthy to be further explored to perfect the immune regulatory system of MDSCs in autoimmune arthritis.

Furthermore, iNOS promotes prolonged survival of cardiac allografts mediated via BM-derived MDSCs (73). Addition of IL-13 to the combination of GM-CSF and G-CSF induced more suppressive BM-derived MDSCs and markedly decreased GVHD lethality, which was correlated with increased production of arg-1 by MDSCs (106). Moreover, MDSCs derived from normal BM cells promoted systemic exhaustion of immune systems conducive to allogenic graft survival under stimulation by IL-6 and GM-CSF (104). However, these induced MDSCs did not affect disease progression of autoimmune diabetes and exhibited paradoxical effects on antigen-specific CD8^+^ T cell proliferation *in vitro* compared with that observed *in vivo*. MDSCs induced by GM-CSF alone, without IL-6, from mouse BM cells induced Th17 cell differentiation independent of MDSC-T cell contact, and this effect was likely related to TGF-β and IL-6 production by MDSCs (51). It is possible that different *in vivo* environments caused the injected MDSCs or complex MDSC subgroups to exhibit different activation states, resulting in distinct immune effects. These results represent challenges in the use of MDSCs but also highlight their possible utility in clinical treatments. One report suggested that MDSCs induced from BM cells under IL-6 combined with GM-CSF and G-CSF were able to effectively ameliorate symptoms in RA mouse models, likely via NO-mediated inhibition of T cell responses (60), and PMN-MDSCs represented the majority of these cells. At present, there are few reports available on the function of induced MDSCs or MDSC subpopulations within the context of autoimmune arthritis. Regarding RA treatment, it may be more valuable to develop efficient induction systems for specific MDSC subpopulations based on their distinct actions within the context of arthritis.

#### Open Questions for Adoptive Transfer of MDSCs

As previously mentioned, the question of whether MDSCs differentiate into mature cells *in vivo* after adoptive transfer, even at sites with a large number of MDSCs, remains unanswered. Some reports have demonstrated the inability of transferred Ly6C^high^ monocytes to develop into DCs under inflammatory conditions (53, 116–118). Nonetheless, osteoclasts that originated from macrophage/monocyte-lineage myeloid precursors similar to MO-MDSCs were activated by multiple inflammatory factors during bone destruction in RA (1). CD11b^+^Gr-1^+^ MDSCs reportedly developed into CtsK^+^ osteoclasts and aggravated bone destruction after transfer into the tibias of CIA mice (59). Another study reported MDSCs induction into osteoclasts *in vitro* under stimulation by various factors responsible for osteoclast differentiation, such as M-CSF and receptor activator for nuclear factor-κ B ligand (RANKL) (119). Accordingly, before these methods are applied in the clinic, it should be addressed whether transferred MDSCs, particularly MO-MDSCs, further differentiate in different inflamed niches of RA patients and whether there are any markers to suggest the dosage or frequency of MDSC injection to avoid overtreatment.

In addition, engineering MDSCs *in vitro* into suppressors that are more potent represents a feasible strategy for the treatment of autoimmune diseases. Induced MDSCs that express self-antigen derived from BM cells *in vitro* effectively ameliorated Ag-induced experimental autoimmune encephalomyelitis (120). Thus, stimulation of MDSCs or precursors using cytokines or antigens to render them more susceptible to or suppressive toward corresponding immune responses represents a promising approach. Whether this strategy is also suitable for RA is unknown, but it should be considered as a potential strategy.

Moreover, many other challenges remain unresolved. For example, how can proliferation be controlled to ensure survival after transfer *in vivo*? Will any side effects occur based on the use of a large number of cells or long-term use? How can these cells be generated in sufficient numbers and properly stored *in vitro* to maintain activity to meet the needs for clinical treatment.

### Prospects for Exosome-Based Therapy

Given the limitations of cell-based therapy, exosome-based biotherapy has recently demonstrated promise for a new treatment strategy (121–124) with fewer side effects compared with drugs and better controllability compared with cell-based therapies. Exosomes, which are secreted by almost all cells and are composed of proteins, RNAs and DNAs under a lipid bilayer structure, are distributed throughout the body (125, 126). In autoimmune arthritis, exosomes are important in the maintenance and progression of arthritic inflammation via active molecule cargo (127, 128). For example, exosomes derived from DCs that express high levels of IL-4 cause remission in established CIA and relieve inflammation in the delayed-type hypersensitivity (DTH) model (129). MDSC-derived exosomes significantly benefit Treg cell expansion and inhibition of T cell responses (130) in alopecia areata models. Moreover, exosomes derived from PMN-MDSCs participate in inhibition of Th1 cell proliferation and promotion of Treg expansion in dextran sulfate sodium (DSS)-induced colitis (123). We also identified a pivotal role for PMN-MDSC-derived exosomes in the remission pathology of CIA mice, mainly by impeding Th17 cell generation.

On the one hand, exosomes retain some characteristics and functions of the donor cells and are easily remolded *in vitro* (125). Accumulating evidence indicates that proteins (126, 131, 132) as well as mRNAs and miRNAs (133, 134) within MDSC-derived exosomes obtained from different disorders are biologically active, and the molecular mechanisms involved are progressively being elucidated (133). Therefore, the use of exosomes directly collected from functional MDSC subpopulations or further reorganized after isolation may address some of the limitations of cell-based therapies. On the other hand, these methodologies may highlight therapeutic pathways for targeting exosomes derived from other cells to dramatically regulate MDSC activity during the pathogenesis of arthritis. Nevertheless, these effects are frequently reported under tumor conditions (135–139), and insufficient data have been reported from autoimmune arthritis microenvironments. Overall, exploring functional small RNAs or proteins within exosomes from MDSCs represents a promising approach for therapeutically targeting autoimmune diseases, and exosomes can be collected or further modified for application in clinical treatments. Regardless, issues regarding the properties of exosomes, such as their biogenesis, composition programming, targeted specificity, life span, and homogeneity, must be addressed prior to clinical application (130).

### Other Immunosuppressive Cell Treatment Options for Autoimmune Diseases

Besides MDSC-based immunosuppressive cell treatments, other suppressive cells such as Tregs, tolerogenic DCs, have also shown promising therapeutic prospects in autoimmune diseases. In order to re-establish the badly damaged balance of T cell subpopulations, three extremely potential strategies for clinical use of Tregs was summarized that expansion of self-antigen-specific natural Tregs *in vivo* such as the promising injection with low-dose IL-2 (140–142), transfer of autogenous antigen-specific natural Tregs after antigen stimulating and propagation *ex vivo*, and transformation of antigen-specific conventional T cells into Tregs *in vivo* or *ex vivo* (143). Similarly, there are some critical problems necessarily to be resolved at the present. For instance, the methodology to transform stable Treg-like epigenetic changes targeting the third part, how to effectively stimulate and induce therapeutic Tregs with self-antigen for the second part, and how to avoid the potential side effects of cytokines promoting Treg cell proliferations after long-time use targing the first. Importantly, there is just one report adoptive transfer of autologous Tregs isolated from a patient with SLE after proliferation *in vitro*, which suggested no significant remission and a dynamic shift from Th1 to Th17 responses although activated Tregs was increased in inflamed skin (144). Therefore, further research and attempt were supposed to be conducted.

Recently, another DC subpopulation, tolerogenic DCs, has shown effectively regulatory effects in immune responses. Increasing evidence suggested that tolerogenic DCs could limit effector T cell responses (145) under signaling stimulations of IL-27 (146), IL-10 (147) or ligands of the aryl hydrocarbon receptor (AhR) (148, 149). In autoimmune arthritis, tolerogenic DCs were found a synergistic suppression with mesenchymal stem cells (MSC) in murine CIA models (150). Moreover, transfer of these cells into organ allograft animal models such as pancreatic islet, small intestine, renal and liver (151). However, it is still unclear that whether tolerogenic DCs was a specific subgroup or just represent a particular activation condition of DCs so far. Therefore, more evidence should be provided to completely recognize the nature, characteristics and roles in immune responses.

Notably, before clinical applications of these immunosuppressive cell treatments, length of curative effects, dose and frequency of injections were supposed to be extremely focused according to experience from preclinical and clinical cytokine treatments (2), which correlated closely with infectious risk and relapse problems. Otherwise, it is likely that internal interactions would be made use of in autoimmune diseases among these suppressive cells such as tolerogenic DCs and Foxp3^+^ Tregs (152, 153), MDSCs and Tregs (66, 73, 98), which suggests a considerable therapeutic strategy by combined transfer application of immunosuppressive cells for clinical treatments of autoimmune diseases.

## Conclusion

Circumstantial findings substantiate the important and controversial roles of MDSC subpopulations in autoimmune arthritis and other inflammation-related diseases (116). Both MDSC subpopulations play important roles in regulating the proliferation, response and differentiation of CD4^+^ T cells during the progression of autoimmune arthritis. However, the maturation potential of these cells should not be ignored. Some reports demonstrate more potent inhibition of arthritis inflammation by PMN-MDSCs compared with MO-MDSCs, and the specific local microenvironment is important for MDSC subpopulations in autoimmune arthritis. Double-edged roles of MDSC subpopulations might depend on inflammatory systems or their various differentiation stages caused by dynamically altered microenvironments during arthritis progression. As exosomes derived from MDSCs are responsible for regulating immune function in immunocytes, exploring potent communication mechanisms of inhibitory MDSC subpopulations to discover more about MDSC-derived exosomes and target functional pathways for the clinical treatment of RNA represent important goals in the coming years.

## Author Contributions

ML wrote the manuscript and discussed the content with the other coauthors. DZ, TW, XX and JT discussed the content with the other coauthors. SW conceived the topic and revised the manuscript.

### Conflict of Interest Statement

The authors declare that the research was conducted in the absence of any commercial or financial relationships that could be construed as a potential conflict of interest.
